# The occurrence of opportunistic pathogenic *Pseudomonas* species in bathing ponds

**DOI:** 10.1007/s12223-024-01229-1

**Published:** 2024-12-05

**Authors:** Dana Baudišová, Šárka Bobková, Petr Pumann

**Affiliations:** National Institute of Public Health, Šrobárova 48/49, 100 42 Prague 10, Czech Republic

**Keywords:** *Pseudomonas otitidis*, *Pseudomonas aeruginosa*, Bathing ponds, Antibiotic resistance, *exoA* gene

## Abstract

The aim of this study is to evaluate opportunistic pathogenic bacteria of the genus *Pseudomonas* in anthropogenically impacted bathing waters, primarily focusing on bathing ponds. The findings include the detection of these bacteria, their susceptibility to selected antibiotics, and the determination of the Exotoxin A *(exoA)* gene using PCR method. *P. aeruginosa* was present in most samples, albeit in low concentrations (1–14 CFU/100 mL). The presence of *P. otitidis*, which is associated with ear infection, in this type of bathing water, was not rare (up to 90 CFU/100 mL). This species would not be detected by the standard methods, including tests on acetamid medium, used for *P. aeruginosa* in water. The isolated strains of *P. otitidis* lack the *exoA* gene and exhibited higher resistance to meropenem compared to *P. aeruginosa*.

**Supplementary Information:**

The online version contains supplementary material available at 10.1007/s12223-024-01229-1.

## Introduction

In the summer months, swimming is a popular activity in the Czech Republic, both in natural environments and in artificial swimming pools. A relatively recent alternative to these “traditional” bathing options are natural swimming ponds, which are fresh water pools where the water is purified solely through natural way. These ponds present also valuable opportunity for research on the impact of anthropogenic pollution, as they are relatively small areas with a high concentration of visitors, with bathers often being the main source of contamination. Among the various potential infections, those caused by bacteria of the genus *Pseudomonas*, mainly *P. aeruginosa* species are of significant concern. *P. aeruginosa* is an opportunistic pathogenic Gram-negative aerobic rod belonging to the Pseudomonadaceae family, typically isolated from aquatic environments. Its increased occurrence in swimming pools, bathing waters, urban water features (e.g., interactive fountains), and swimming ponds may be associated with an increased risk of mucosal inflammation (e.g., external ear canal conjunctivitis, nasal canal mucosa, abrasions, and other open skin injuries) or urinary infections in more susceptible individuals (Seyfries and Cook 1984; van Asperen et al. 1995). *P. otitidis*, a related species described in 2006, has been isolated from clinical material (Clark et al. 2006) and is primarily associated with ear infections, though cases of non-otitis infections have also been reported (Caixintha et al. 2021). Further work has shown that this species can also cause skin infections. Despite their close relationship, *P*. *aeruginosa* and *P*. *otitidis* can be well distinguished by matrix-assisted laser desorption ionization–time of flight mass spectrometry (MALDI-TOF MS). However, it is likely that *P*. *otitidis* infections have been underestimated, as they may have been misidentified as *P*. *aeruginosa* (Caixintha et al. 2021). To date, the occurrence of *P*. *otitidis* species in environmental samples and also in bathing waters (including bathing ponds) has been rarely studied. In addition to being opportunistic pathogenic, both species have been described to have antibiotic resistance (including multidrug resistance), particularly to carbapenems (Milligan et al. 2023; Thaller et al. 2011; Kolář et al. 2013; Logan et al. 2017). The original aim of this short communication was to optimize a method for the determination of *P. aeruginosa* in anthropogenically influenced bathing waters (samples with high levels of background microflora) and to assess the level of contamination of bathing ponds with this opportunistic pathogen. However, as we began isolating another opportunistic pathogen, *P*. *otitidis*, in addition to *P*. *aeruginosa*, we shifted our focus to include also this species. In addition to quantifying both opportunistic pathogenic pseudomonades, we evaluated biochemical characteristics, which are used in the confirmation of *P*. *aeruginosa*, if they would be useful also for the confirmation of *P*. *otitidis*. We also assessed the susceptibility of isolated strains to selected antibiotics and determined the presence of the Exotoxin A gene (*exoA*), which is associated with pathogenicity and may help to distinguish between virulent and non-virulent strains.

## Methodology

### Sampling

Water samples were collected during the summer of 2023 (from June to September) from four locations of bathing ponds in Prague and Central Bohemia, two of which were sampled repeatedly. The repeatedly sampled sites experienced high visitor numbers on hot summer days with maximum capacity ranging from 600 to 1000 people at a time, typically reaching full occupancy. Grab samples were collected, always 1–2 samples per site, depending on the area. To capture additional strains for studying ATB resistance and the *exoA* gene, samples were also collected from three urban water fountains. A total of 23 water samples were collected and analyzed. The control strains used included a collection strain of *P. aeruginosa* CCM 1960, a strain of *P. aeruginosa* isolated in 2018 from a hotel swimming pool/whirpool in the Pardubice region, where it caused an outbreak of ear infections and collection strain *P. otitidis* CNCTC 8175.

### Laboratory determination of *E.* coli and intestinal enterococci

*E. coli* was determined by β-D-glucuronidase enzyme activity by the Colilert18 Quanti/TRAY method (IDEXX). Intestinal enterococci were determined by membrane filtration and culture on agar according to Slanetz and Bartley (48 h at 36 °C) and confirmation on Bile aesculine azide agar (2 h at 44 °C). A volume of 100-mL sample was processed.

### Laboratory determination of *P.aeruginosa*

To detect opportunistic pathogenic pseudomonades, the standard method according to EN ISO 16266 (n.d.) was used which was modified to eliminate the background microflora. Water samples (100 mL) were filtered through a 0.45-µm porosity membrane filter and transferred to *Pseudomonas* (CN) agar. Cultivation was carried out for 4 h at 36 °C followed by cultivation for 48 h at 44 °C. At the end of this cultivation, colonies showing light green fluorescence were counted and labeled, and the plated membrane filters were placed in 36 °C for an additional 24 h (it can appear the formation of the light green pigment pyocyanine, which is not formed at 44 °C). Acetamide and King’s medium were used as further confirmatory tests and the strains were subsequently identified by MALDI-TOF MS (Brueker Diagnostics). The confirmation at the score levels above 2000 were accepted.

### Antibiotic resistance tests

To determine antibiotic resistance (sensitive, resistant, and i-strains, i.e., sensitive strains at increased exposure) isolated and identified strains were tested by the cultivation method (plate diffusion method) according to the European (2023) Committee on antimicrobial susceptibility testing; clinical breakpoints were taken from Table 13.1, valid from 29. 6. 2023. Used discs (Oxoid) with antibiotics: piperacillin/tazobactam (30/6 µg), ceftazidime (10 µg), meropenem (10 µg), imipenem (10 µg), ciprofloxacin (5 µg), and amikacin (30 µg).

### PCR amplification of part of the *exoA* gene

The *exoA* gene was determined by PCR amplification of a 397 bp *exoA* gene portion. The amplification conditions were based on the publication by Khan and Cerniglia (1994) using Taq Purple DNA polymerase (TopBio). The PCR reaction was performed on a qTowerG instrument (Analytikjena). 10 µL of the PCR mixture was subsequently analyzed on a 1.5% agarose gel (5 V/cm) containing GelRed and visualized under UV light. Selected PCR products were verified by Sanger sequencing performed by SeqMe.

## Results

### Detection of *P.aeruginosa* and *P.otitidis*

**Fig. 1:**
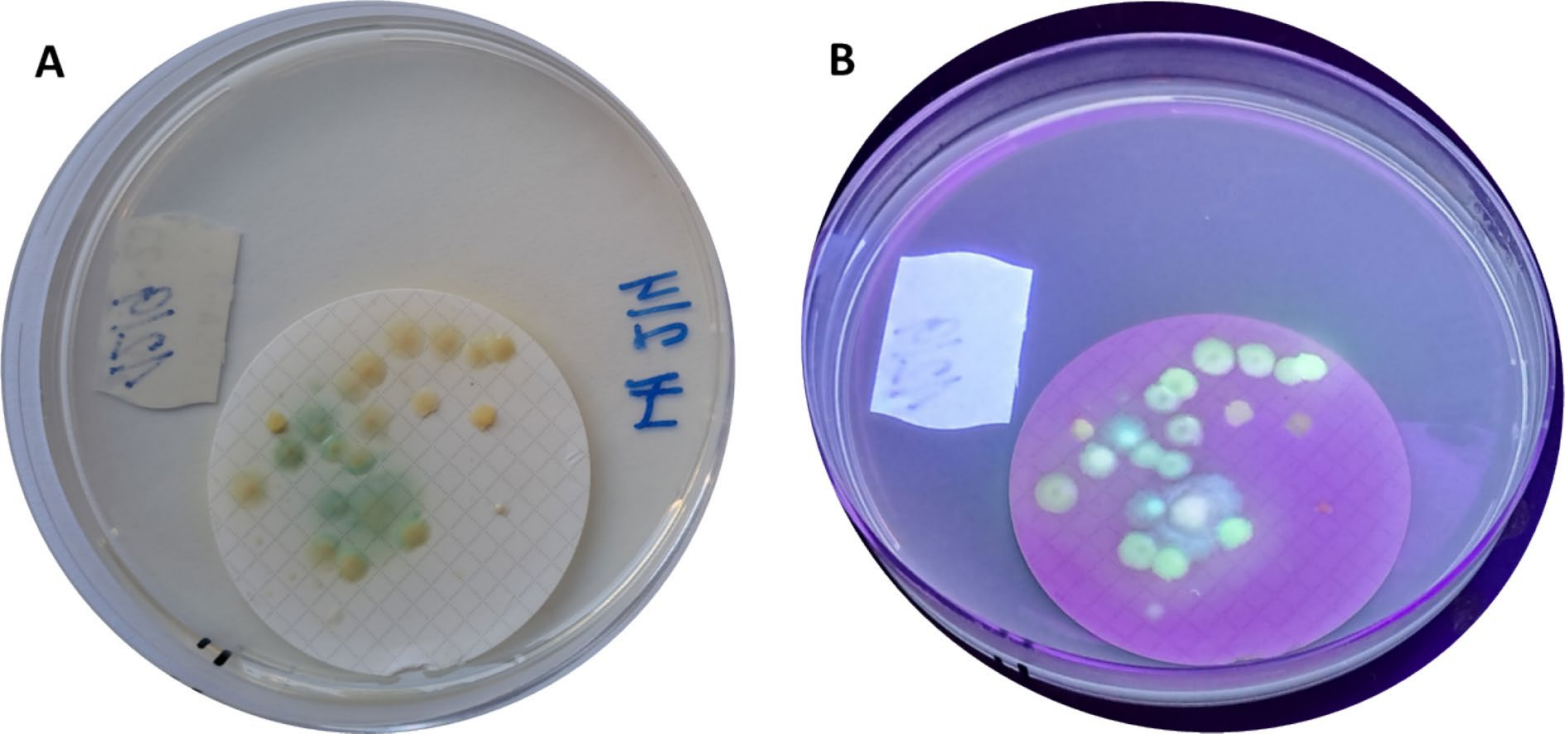
Colonies of opportunistic pathogenic pseudomonades on *Pseudomonas* CN agar (**A**), under UV lamp at 254 nm (**B**)

The modified method used for the determination of *P. aeruginosa* eliminated most of the background microflora, allowing for accurate isolation and counting of the colonies. *P. aeruginosa* formed larger, blue-green colonies, whereas *P. otitidis* formed yellowish colonies with noticeable fluorescence (Fig. 1). Among the confirmatory tests for *P. aeruginosa*, only the reaction on King’s medium was positive for *P. otitidis*. Additionally, 90% of *P. otitidis* strains exhibited a negative reaction on acetamide medium, and the hydrolysis of the 7-amino-4-methylcoumarin aminopeptidase substrate in selective medium was only very weak. Therefore, *P. otitidis* cannot be detected in routine laboratory testing by EN ISO 16266.

### Isolate identification

A total of 56 strains of pseudomonades were species-determined by MALDI-TOF MS (some of them rather tentative; see Table S1): 42 strains of *P. aeruginosa* and 14 strains of *P. otitidis*. The deposition procedure is in progress in the case of isolate *P. otitidis* PA33 (CNCTC collection number 8216).

### Occurrence of opportunistic pathogen *Pseudomonas* species in bathing ponds

In addition to *P. aeruginosa* and *P. otitidis*, the standard fecal contamination indicators *E. coli* and intestinal enterococci were determined in the samples. The results are presented in Table 1. Only 2 samples from the sampling site D were *P. aeruginosa* negative.

**Table 1:** Numbers (arithmetic mean and range of values obtained) of fecal pollution indicators, *P. aeruginosa* and *P. otitidis* at the studied bathing ponds (CFU/100 mL). For site A, the mean value is that obtained (1 sample). For *P. otitidis*, mean values are not given because their values varied widely

	A (*n* = 1)	B (*n* = 2)	C (*n* = 8)	D (*n* = 8)
*E. coli* mean value	22	16	33	27
*E. coli* range	-	12–19	6–105	6–96
Enterococci mean value	18	9	7	10
Enterococci range	-	3–15	3–20	2–27
*P. aeruginosa* mean value	1	1	4	5
*P. aeruginosa* range	-	1–1	1–8	0–14
*P. otitidis* range	1	1–1	0–15	0–90

### Antibiotic resistance tests

Antibiotic resistance was tested for strains from all positive samples, with a maximum of six strains per sample. All strains were sensitive to ceftazidime, and the majority were sensitive to amikacin, including all *P. otitidis* and 58% of *P. aeruginosa* strains. Resistance was observed with meropenem, particularly in *P. otitidis*, where 71.4% of the strains were resistant, compared to only 7% of the *P. aeruginosa* strains. The meropenem resistance was also confirmed in the reference strain CNCTC 8175. Since clinical breakpoints for *P. otitidis* are not defined, those for *P. aeruginosa* were taken for evaluation. The remaining strains (and antibiotics) were classified as i-strains (sensitive at increased exposure) according to the European Committee on antimicrobial susceptibility testing; clinical breakpoints were taken from Table 13.1, valid from 29. 6. 2023.

### PCR amplification of *exoA*

**Fig. 2:**
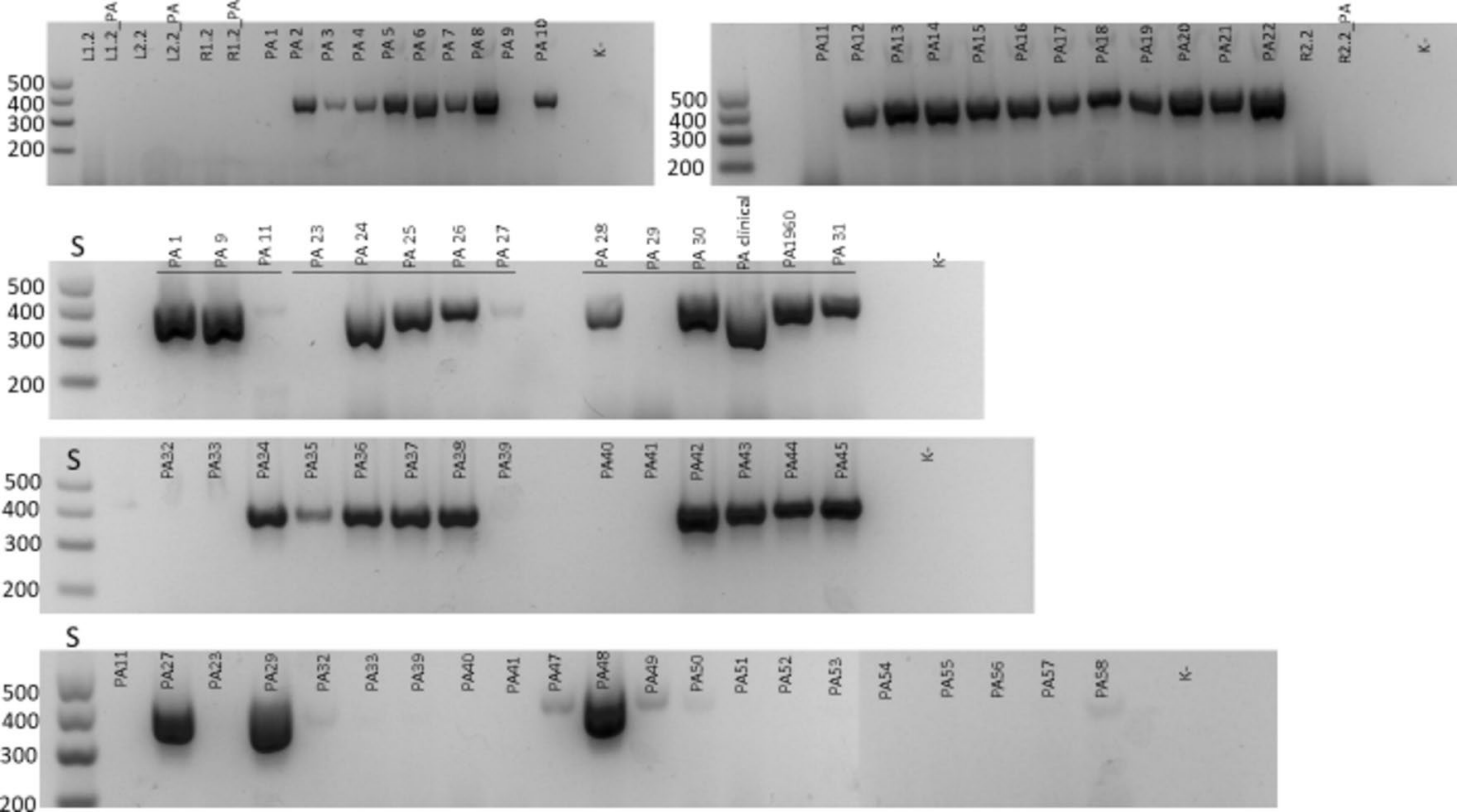
Results of PCR amplification of part of the *exoA* gene**.** 1.5% agarose gel. S: standard molecular weights. In case the PCR was negative, the reaction was repeated once more with newly isolated DNA, therefore several strains (e.g., 11, 9, 23, and 27) are shown in two different gels. Numbers correspond to the numbers listed in table S1. Samples L1.2,…, R 2.2 respond to PCR reaction were in the whole water samples (without DNA isolation) was used for the PCR reaction. This approach did not work; therefore, we did not use it further

All strains (56 strains isolated and 3 control strains) were also tested for the presence of part of the *exoA* gene. All 42 *P. aeruginosa* strains contained *exoA* as indicated by successful amplification with the primers used. In contrast, 12 of the 14 *P. otitidis* strains were negative, showing no amplification (Figs. 2 and S1). The remaining two *P. otitidis* strains were initially positive for *exoA*; however, the amplification results were not consistently clear in repeated PCR reactions. Subsequent sequencing of the PCR products revealed that these strains likely had non-specifically bound primers targeting a different part of the chromosome, leading to the amplification of a different random region. As a result, all *P. otitidis* strains were determined to be *exoA* negative, as no reliable amplification was observed.

## Discussion

Although the bathing ponds studied were significantly influenced by humans (the number of visitors reached repeatedly up to 1000 visitors at a time), the values of fecal contamination indicators, *E. coli* and enterococci, almost always met the legislative requirements (Decree No. 238/20011 Coll. (n.d.): 100 and 50 CFU/100 mL, respectively). However, *P. aeruginosa* was detected in nearly every sample, though typically in low concentrations (mostly up to 10 CFU/100 mL).

Although the optimization of the method was originally aimed to improve the detection of *P. aeruginosa* by eliminating the background microflora, it had a significant unintended consequence. We were able to repeatedly isolate the opportunistic pathogenic species, *P. otitidis*, which is rarely detected in environmental samples and may cause health problems in such heavily frequented sites. Elevated numbers (15–80 CFU/100 mL) of *P. otitidis* were also detected in 6 samples from the sites C and D, collected during the second part of the summer season after large crowds visited the bathing ponds, reaching their upper capacity limits. *P. otitidis* has also been detected in geothermal bathing pools in Iceland (Thorolfsdottir and Marteinsson 2013). This species cannot be detected by the standard *P. aeruginosa* method EN ISO 16266 and is not expected to be included among the monitored indicators in the foreseeable future. However, its frequent presence in bathing waters can lead to infections, particularly in immunocompromised individuals. An additional analysis of virulent strains of pseudomonades could involve detecting the presence of the *exoA* gene. However, we did not detect this gene in *P. otitidis*. Khan and Cerniglia (1994) similarly found no evidence of the *exoA* gene even in other *Pseudomonas* species, suggesting that this gene is exclusively associated with *P. aeruginosa*. Moreover, direct detection of pseudomonades by PCR methods from the environment with a high background microflora is challenging due to the relatively low abundance of the target group compared to the total bacterial population. While methods such as MALDI-TOF MS and PCR are very convenient for confirmation, we do not expect their incorporation to EN ISO 16266.

Resistance of *P. otitidis* strains to meropenem has already been published (Thaller et al. 2011). Although the number of strains we tested was relatively small, making direct comparisons to literature data less definitive, our findings suggest that the strains isolated from bathing waters exhibit lower resistance than those from hospital settings or clinical material (Kolář et al. 2013). They reported that, between 2008 and 2013, 30–50% strains of *P. aeruginosa* isolates from hospitalized patients’ clinical material was resistant to meropenem, whereas we detected only 7% resistance among our *P. aeruginosa* strains.

## Conclusions

Although *P. aeruginosa* was detected in most of the samples, the numbers detected were not high (up to 10 CFU/100 mL). In addition to *P. aeruginosa*, *P. otitidis*, which was primarily isolated from clinical material as an ear pathogen, was also detected. This bacterium exhibited a negative acetamide test and showed weak hydrolysis of the 7-amino-4-methylcoumarin aminopeptidase substrate, meaning it cannot be detected by standard method for *P. aeruginosa* according to EN ISO 16266. *P. otitidis* was detected in higher concentrations during the second half of the swimming season, particularly after large crowds visited the swimming ponds, reaching their upper capacity limit. Its presence can account for infection, especially in immunocompromised individuals.

## Supplementary Information

Below is the link to the electronic supplementary material.

Supplementary file1 (DOCX 78 KB)
