# Supplementary material for: The occurrence of opportunistic pathogenic Pseudomonas species in bathing ponds
Source: Folia Microbiol (Praha). 2024 Dec 5;70(1):253–7. doi: 10.1007/s12223-024-01229-1 (PMC11861126; doi:10.1007/s12223-024-01229-1)
Supplement: Supplementary file 1 — Supplementary file1 (DOCX 78 KB) [file 12223_2024_1229_MOESM1_ESM.docx]

Suplementary data.:
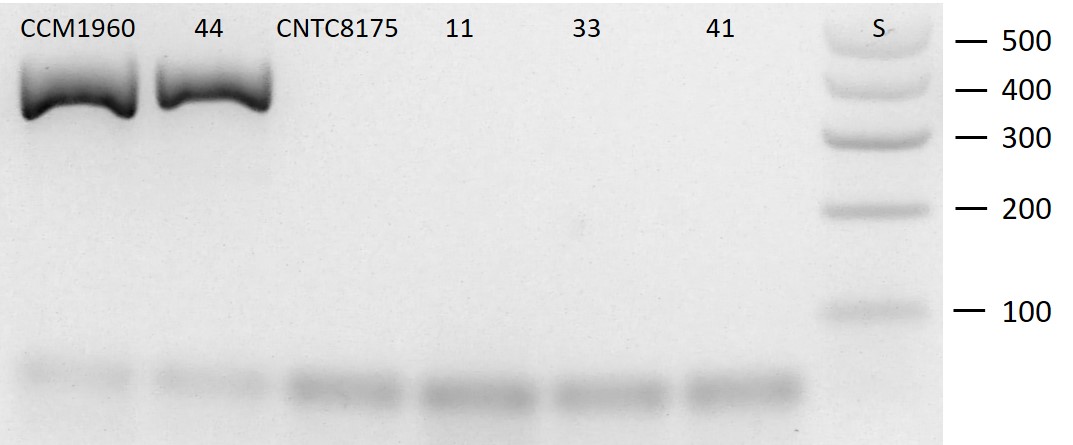


Figure S1: Supplementary results of PCR amplification of part of the *exoA* gene**.** 1.5% agarose gel. S - Standard molecular weights. Numbers correspond to the numbers listed in table S1. CCM 1960 is the reference strain for *P. aeruginosa* and CNTC8175 is the reference strain for P. *otitidis*. The strain No 44 is *P. aeruginosa,* and strains No 11,33 and 41 are *P. otitidis.*

Table S1: List of tested strains, their identification by MALDI-TOF MS and the results of PCR amplification of the *exoA* gene. *P. otitidis* are in bold and the two samples with unclear results are grey.

| **Strain No.** | **Bathing pond (A,B,C,D) and cities fountains CFA CFB,CFC) /Exact date of isolation (2023)** | **Species MALDI-TOF MS identification** | **MALDI-TOF MS identification score** | **PCR exo A** |
| --- | --- | --- | --- | --- |
| PA2 | A/31.7. | aeruginosa | 2.30 | + |
| PA3 | CFA/10.7. | aeruginosa | 2.19 | + |
| PA4 | CFA/10.7. | aeruginosa | 2.05 | + |
| PA5 | CFB/24.7. | aeruginosa | 2.27 | + |
| PA6 | CFB/24.7. | aeruginosa | 2.23 | + |
| PA7 | CFB/24.7. | aeruginosa | 2.35 | + |
| PA8 | CFB/24.7. | aeruginosa | 2.28 | + |
| PA9 | B/24.7. | aeruginosa | 2.31 | + |
| PA10 | B/24.7. | aeruginosa | 2.21 | + |
| **PA11** | B/24.7. | **otitidis** | 2.33 | **-** |
| PA12 | C/19.6. | aeruginosa | 2.12 | + |
| PA13 | C/19.6. | aeruginosa | 2.31 | + |
| PA14 | C/17.7. | aeruginosa | 2.34 | + |
| PA15 | C/17.7. | aeruginosa | 2,.27 | + |
| PA16 | C/17.7. | aeruginosa | 2.21 | + |
| PA17 | D/19.6. | aeruginosa | 2.36 | + |
| PA18 | D/17.7. | aeruginosa | 2.24 | + |
| PA19 | D/17.7. | aeruginosa | 2.45 | + |
| PA20 | D/17.7. | aeruginosa | 2.35 | + |
| PA21 | D/17.7. | aeruginosa | 2.33 | + |
| PA22 | D/19.6. | aeruginosa | 2.11 | + |
| **PA23** | B/24.7. | **otitidis** | 2.00 | +/- |
| PA24 | C/19.6. | aeruginosa | 2.30 | + |
| PA25 | C/17.7. | aeruginosa | 2.40 | + |
| PA26 | CFA/10.7. | aeruginosa | 2.13 | + |
| PA27 | D/19.6. | aeruginosa | 2.37 | + |
| PA28 | D/19.6. | aeruginosa | 2.42 | + |
| PA29 | D/19.6. | aeruginosa | 2.32 | + |
| PA30 | D/19.6. | aeruginosa | 2.38 | + |
| PA31 | CFC/24.7. | aeruginosa | 2.27 | + |
| **PA32** | **D/22.8.** | **otitidis** | 1.79 | **-** |
| **PA33**/future CNCTC 8216 | **D/22.8.** | **otitidis** | 2.32 | **-** |
| PA34 | D/22.8. | aeruginosa | 2.46 | + |
| PA35 | D/22.8. | aeruginosa | 2.42 | + |
| PA36 | D/22.8. | aeruginosa | 2.27 | + |
| PA37 | D/22.8. | aeruginosa | 2.37 | + |
| PA38 | D/22.8. | aeruginosa | 2.36 | + |
| **PA39** | **C/22.8.** | **otitidis** | 1.74 | **-** |
| **PA40** | **C/22.8.** | **otitidis** | 1.81 | **-** |
| **PA41** | **C/22.8.** | **otitidis** | 2.08 | **-** |
| PA42 | C/22.8. | aeruginosa | 2.31 | + |
| PA43 | C/22.8. | aeruginosa | 2.32 | + |
| PA44 | C/22.8. | aeruginosa | 2.24 | + |
| PA45 | C/22.8. | aeruginosa | 2.11 | + |
| PA46 | C/22.8. | aeruginosa | 2.18 | + |
| PA47 | C/22.8. | aeruginosa | 2.03 | + |
| PA48 | C/11.9. | aeruginosa | 2.34 | + |
| PA49 | C/11.9. | aeruginosa | 1.83 | + |
| **PA50** | **C/11.9.** | **otitidis** | 1.79 | +/- |
| **PA51** | **C/11.9.** | **otitidis** | 1.84 | **-** |
| **PA52** | **C/11.9.** | **otitidis** | 1.82 | **-** |
| PA53 | C/11.9. | aeruginosa | 2.43 | **-** |
| **PA55** | **D/11.9.** | **otitidis** | 2.02 | **-** |
| **PA56** | **D/11.9.** | **otitidis** | 1.99 | **-** |
| **PA57** | **D/11.9.** | **otitidis** | 2.03 | **-** |
| **PA58** | **D/11.9.** | **otitidis** | 1.96 | **-** |
| clinical sample |  | aeruginosa | 2.28 | + |
| PA 1960 |  | aeruginosa from Czech Culture collection (CCM) | 2.31 | + |
| PA 8175 |  | **otitidis** from CNCTC | 2.05 |  |
